# GTS-21 has cell-specific anti-inflammatory effects independent of α7 nicotinic acetylcholine receptors

**DOI:** 10.1371/journal.pone.0214942

**Published:** 2019-04-04

**Authors:** Brijesh K. Garg, Ralph H. Loring

**Affiliations:** Department of Pharmaceutical Science, Northeastern University, Boston, Massachusetts, United States of America; INSERM, FRANCE

## Abstract

α7 Nicotinic acetylcholine receptors (nAChRs) reportedly reduce inflammation by blocking effects of the important pro-inflammatory transcription factor, nuclear factor kappa-light chain-enhancer of B cells (NFκB). The α7 nAChR partial agonist GTS-21 reduces secretion of pro-inflammatory cytokines including interleukin-6 (IL6) and tumor-necrosis factor (TNF) in models of endotoxemia and sepsis, and its anti-inflammatory effects are widely ascribed to α7 nAChR activation. However, mechanistic details of α7 nAChR involvement in GTS-21 effects on inflammatory pathways remain unclear. Here, we investigate how GTS-21 acts in two cell systems including the non-immune rat pituitary cell line GH4C1 expressing an NFκB-driven reporter gene and cytokine secretion by *ex vivo* cultures of primary mouse macrophages activated by lipopolysaccharide (LPS). GTS-21 does not change TNF-stimulated NFκB signaling in GH4C1 cells expressing rat α7 nAChRs, suggesting that GTS-21 requires additional unidentified factors besides α7 nAChR expression to allow anti-inflammatory effects in these cells. In contrast, GTS-21 dose-dependently suppresses LPS-induced IL6 and TNF secretion in primary mouse macrophages endogenously expressing α7 nAChRs. GTS-21 also blocks TNF-induced phosphorylation of NFκB inhibitor alpha (IκBα), an important intermediary in NFκB signaling. However, α7 antagonists methyllycaconitine and α-bungarotoxin only partially reverse GTS-21 blockade of IL6 and TNF secretion. Further, GTS-21 significantly inhibited LPS-induced IL6 and TNF secretion in macrophages isolated from knockout mice lacking α7 nAChRs. These data indicate that even though a discrete component of the anti-inflammatory effects of GTS-21 requires expression of α7 nAChRs in macrophages, GTS-21 also has anti-inflammatory effects independent of these receptors depending on the cellular context.

## Introduction

Chronic inflammation may lead to several inflammatory disorders including sepsis, rheumatoid arthritis, asthma, diabetes and Crohn’s disease [1, 2] and involves production and secretion of various pro-inflammatory cytokines [3] including IL6, TNF and high mobility group box-1 (HMGB1). Koopman et al. [4] recently proposed vagus nerve stimulation as therapy against the inflammation found in rheumatoid arthritis, based in part on previous work [5] that vagus nerve stimulation requires cholinergic activation to prevent TNF secretion in response to endotoxins, such as lipopolysaccharide (LPS). LPS is a gram-negative bacteria cell wall component that activates NFκB-mediated inflammatory signaling in cells expressing Toll-like 4 receptors (TLR4) [6]. Later, Wang et al. [7] reported that activated α7 nicotinic acetylcholine receptors (nAChRs) inhibit LPS-induced pro-inflammatory cytokine secretions without affecting anti-inflammatory mediators such as IL-10. Wang et al. [8] found that α7 nAChRs mediate anti-inflammatory signaling in part by blocking NFκB activation in a mouse sepsis model. Follow-up studies have since established that during macrophage activation, the afferent vagus nerve senses pro-inflammatory mediators in the periphery to relay the message to the brain which releases acetylcholine via efferent vagus activity to inhibit inflammation locally through α7 nAChRs activation [9]. This pathway is known as the ‘cholinergic anti-inflammatory pathway’ [9, 10] and has increased interest in α7 nAChR agonists, such as GTS-21, as potential anti-inflammatory drugs.

α7 nAChRs are pentameric ion-channel receptors involved in memory and cognition and are implicated in various disorders including Alzheimer’s, Parkinson’s, schizophrenia, cystic fibrosis and inflammation [7, 11–15]. These receptors are found throughout human brain and the peripheral nervous system. Also, these receptors have been observed recently on certain immune cells such as macrophages and lymphocytes [16], and α-bungarotoxin (αBGT), a snake neurotoxin relatively specific for α7 nAChRs, shows the possible presence of these receptors on macrophage cells [7]. *In vivo* animal models of inflammation suggest that nicotine, like ACh, can reduce pro-inflammatory cytokines and even improve survival in mouse models of endotoxemia and sepsis [8, 17–21]. However, nicotine lacks receptor specificity and possesses major toxicity challenges [22, 23]. Hence, recent studies have focused on α7 nAChR-selective ligands to find an anti-inflammatory drug devoid of nicotine-like toxic side-effects, including GTS-21, 3-(2,4-dimethoxybenzylidene) anabaseine, a partial α7 nAChR agonist [24].

GTS-21 (also known as DMBX-anabaseine) inhibits pro-inflammatory cytokines like TNF, IL-1β, IL6 and HMGB1 and improves survival in murine endotoxemia and sepsis models [8, 24–27]. Further, its anti-inflammatory effects are widely ascribed to actions as a partial α7 nAChR agonist. However, Nullens et al. [28] found that GTS-21 decreased systemic and colonic levels of IL-6, colonic permeability and levels of infection in both wild-type (WT) and α7 knockout septic mice. These *in vivo* results suggest that GTS-21 has anti-inflammatory effects that are independent of its effects as a partial α7 agonist. We sought to investigate GTS-21 effects at a molecular level in macrophages derived from both WT and alpha7 knockout animals. We also investigated whether GTS-21 causes anti-inflammatory signaling in rat GH4C1 cells, a pituitary-derived cell line transfected with α7 nAChR. GH4C1 cells do not express mRNA for known nicotinic receptor α subunits [29] but readily express electrophysiologically-functional receptors when transfected with the α7 nAChR gene [30]. Our results suggest that α7 nAChR expression in the heterologous cell line does not suppress inflammatory signaling and that GTS-21 has both α7 nAChR-dependent and α7 nAChR-independent anti-inflammatory effects in mouse macrophages.

## Materials and methods

### Cell cultures and animals

All studies involving mice were approved by Northeastern University’s Institutional Animal Care and Use Committee (NU-IACUC). Northeastern’s animal care and use program holds an assurance with the Office of Laboratory Animal Welfare (OLAW) and is accredited by the Association for the Assessment and Accreditation for Laboratory Animal Care (AAALAC). In our NU-IACUC approved protocol, 15-0522R-“Nicotinic anti-inflammatory effects in mouse macrophages”, mice were injected intraperitoneally with sterile thioglycolate and euthanized four days later by CO_2_ inhalation followed by cervical dislocation in accordance with the American Veterinary Medical Association Guidelines for Euthanasia of Animals. All efforts were made to minimize suffering. GH4C1 rat pituitary cells were obtained from American Type Culture Collection (ATCC, Manassas, VA) and grown at 37°C in 5% CO_2_. GH4C1 cells were grown in F-10 complete growth medium: Ham’s F-10 basic medium containing 1% penicillin-streptomycin (both from Thermo-Fisher) supplemented with 10% fetal bovine serum (Premium Select from Atlanta Biologicals, Lawrenceville, GA). Male C57BL/6 wild type and α7 nAChR knockout mice (8–12 weeks old; purchased from Jackson Laboratory, Bar Harbor, Maine) were housed under standard conditions in an Institutional Animal Care and Use Committee approved animal facility. Animals were provided with food and water *ad libitum*. Mouse primary macrophages were isolated from peritoneal cavities as described in Zhang et al. [31]. Briefly, 1ml sterile thioglycollate medium (Fisher Scientific catalog #L21199) was injected into mouse peritoneal cavities, the liquid contents of the cavities were harvested four days later, and the resulting cells were grown in DMEM complete growth medium, consisting of DMEM basic medium containing 1% penicillin-streptomycin (both from Thermo-Fisher) supplemented with 10% fetal bovine serum (Hyclone from GE Healthcare Life Sciences, Pittsburgh, PA).

### Plasmids and RNA

Total GH4C1 and primary macrophage RNA was extracted using a TRIzol Plus RNA purification kit (Invitrogen, Carlsbad, CA), and quantified with a NanoDrop ND-1000 UV-Vis spectrophotometer. Complementary DNA (cDNA) synthesis from 1 μg of total RNA for each reaction was carried out using the AffinityScript^TM^ QPCR cDNA synthesis kit, (Agilent Technologies, Santa Clara CA). α7 nAChR primers (5’: ACATGTCTGAGTACCCCGGA and 3’: AGGACCACCCTCCATAGGAC) were found using Pubmed primer–BLAST (NCBI, Bethesda MD) and obtained from Integrated DNA Technologies (Coralville, IA). The primers were designed to amplify both mouse and rat α7 nAChR cDNA using 32 cycles of 95 ^0^C denaturation (30 sec, 5 min before first cycle), 57.5°C annealing (30 sec) and 68°C extension steps (1 min) to get an expected 264 bp amplicon. Approximately 100 ng of cDNA (equivalent to 1/10^th^ of the starting RNA amount) was used to perform polymerase chain reaction (PCR) analysis using Platinum Taq polymerase (Invitrogen, Carlsbad, CA). PCR product was analyzed by gel electrophoresis on a 1.5% agarose gel and sequenced by Genewiz (Cambridge, MA). The full-length rat α7 nAChR sequence cloned into Invitrogen pRep4 plasmids have been previously described (Lee et al., 2009). pEGFP-N1 was obtained from Clontech, Mountain View, CA. NFκB reporter assays were performed using the PNiFty-SEAP plasmid (Invivogen, San Diego, CA., SEAP being a SErcreted Alkaline Phosphatase reporter gene). The NFκB promoter element together with the SEAP open reading frame was excised from this plasmid and inserted into an episomal pRep9 plasmid (Invitrogen) modified to have blasticidin antibiotic resistance (P9KB, the final sequence is available upon request).

### Reagents and antibodies

α-Bungarotoxin (αBGT) was obtained from Biotoxins Inc., St Cloud, FL, and radioiodinated using iodogen (Pierce Chemical, Rockford, IL) as previously described [32]. Rat TNF (catalog# CYT-393) was purchased from ProSpecbio, East Brunswick, NJ. GTS-21 (# SML0326), Pyrrolidine dithiocarbamate (PDTC, # P8765), methyllycaconitine citrate (MLA, # M168), and LPS (# L6529) were purchased from Sigma Aldrich, St. Louis, MO. Rabbit polyclonal anti-GAPDH (# PA1-988) and monoclonal anti-phospho-IκBα pSer32 (#PIMA515087) were obtained from Thermo-Fisher Scientific, Waltham, MA. Alexa Fluor 488 anti-mouse/human CD11b #101219 and Alexa Fluor 647 anti-mouse F4/80 Antibody #123121 were purchased from BioLegend, San Diego, CA. Secondary HRP-conjugated anti-rabbit (# 7074) antibody was purchased from Cell Signaling Technology and used at 1:1000 dilution.

### Transfections

GH4C1 cells were transfected as previously described [33]. Briefly, cells were plated at 500,000 cells/well in a BD Falcon 6-well plate in F-10 complete growth medium 24 h prior to transfection with lipofectamine LTX (Invitrogen, Carlsbad, CA). Cells were serum starved (1 ml F-10 medium only) for 1 h before adding transfection reagents consisting of 3 μg plasmid DNA, 9 μl lipofectamine LTX and 3 μl Plus reagent per well in 300 μl reduced serum Opti-MEM (Invitrogen) using green fluorescent protein (pEGFP-N1) as a control transfection. Four hr post-transfection, 1 ml complete growth medium was added to make the final volume of 2 ml/well. Next day, supernatant medium was replaced by complete growth medium to maintain good cell viability. Transfection efficiency was monitored by fluorescence microscopy 48–72 hr post-transfection. Three days later, α7 nAChR transfected cells were selected with hygromycin (100 μg/ml, Invivogen, San Diego CA), and NFκB-SEAP transfected cells were selected with blasticidin (10 μg/ml, Invivogen). Cells transfected with both α7 nAChR and NFκB-SEAP were selected with both 100 μg/ml hygromycin and 10 μg/ml blasticidin. Transfected cells were cultured and used for PCR analysis, ^125^I-αBGT binding studies, and SEAP experiments.

### Western blots

For pIκB western blots, cells were grown in a 6-well BD Falcon plate and were pretreated for 30 min with different concentrations of GTS-21 (followed by 1 h treatment with TNF or LPS) and washed with ice-cold phosphate buffered salineon the experimental day. After washing, cells were immediately scraped off the surface and centrifuged at 12,000 g at 4°C for 10 min. Supernatant was discarded, and the cells were resuspended in radio-immunoprecipitation assay (RIPA) lysis buffer (150 mM NaCl, 20 mM Tris, 1% NP-40, 0.1% sodium dodecyl sulfate, 1 mM EDTA, pH 8.0) containing Halt protease inhibitor cocktail (Thermo Fischer Scientific, Waltham, MA). Cell suspensions were sonicated for 10–15 seconds to ensure cell lysis. Cell suspensions were kept on ice for 30–40 min and vortexed every 5–10 min before centrifugation at 9600 g at 4°C for 10 min. Supernatants were collected in a fresh 1.5 ml centrifuge tube, and total protein was quantified using a Pierce BCA protein assay kit. Cell lysates were reduced using Bolt 1X lithium dodecyl sulfate loading buffer containing 0.1 M dithiothreitol from Thermo Fisher Scientific, followed by heating the samples at 70°C for 10 min. Samples were kept on ice for 5 min before 40 μg total protein of each sample was loaded in each well on a Bolt^TM^ 4–12% Bis-Tris protein gel for protein separation. Manufacturer’s recommended running and transfer buffers were used with the iBlot 2 dry blotting system to transfer the protein onto nitrocellulose. Blocking was done with 5% bovine serum albumin (BSA) containing tris-buffered saline with 0.1% Tween-20 (TBS-T); the membrane was incubated in primary antibody solution (made in 5% BSA at recommended dilution) overnight at 4°C on a rocker. On the following day, the blot was washed with TBS-T 3–5 times and incubated with secondary antibody conjugated to horseradish peroxidase (HRP) at 1:1000 dilution for 1 h. Protein-antibody complex was visualized on the blot using SuperSignal West Pico Chemiluminescent substrate (Thermo Scientific) and the image was captured by a ChemiDoc XRS Imager from Biorad. GAPDH immunostaining was performed on each blot to confirm equivalent protein loading across the wells.

### Enzyme-linked immunosorbent assay (ELISA)

Primary mouse macrophages were plated at 50,000 cells/well in a 96-well plate 24 h prior to drug treatment. The following day, cells were pretreated with various concentrations of GTS-21 for 30 min before bringing the final volume to 200 μl/well with or without 10 ng/ml LPS. Sixteen h after LPS treatment, TNF and IL6 ELISAs were performed on 20 μl samples as per manufacturer’s instructions (eBioscience, San Diego, CA catalog#s 88-7324-22 (TNF), 88-7064-88 (IL6)). When used, pretreatments with αBGT or MLA were 1 h. Absorbances were read at 630 nm on a BioTek Synergy plate reader (Winooski, VT).

### [^125^I]-labeled-αBGT binding assay

Radioactive binding assays were performed to detect surface α7 nAChR expression as previously described [34]. Cells were plated at 200,000 density per well in a 24-well plate on day 1. ^125^I-αBGT binding assay was performed when cells were 80% confluent. Cells were washed three times with sodium bicarbonate and 0.1% BSA containing Hank’s balanced salt solution (HBSS). BSA washing was used to reduce non-specific binding. Cells were incubated with 10 nM ^125^I-αBGT (unless stated otherwise) for 3 h at 4°C to measure total surface binding. Non-specific binding was determined by the addition of 1μM αBGT. After washing the cells three times in HBSS + BSA, cells were lysed for 10–15 minutes on ice by the addition of 100 μl extraction buffer (0.5 M NaOH + 1% Triton X-100). Lysates were transferred into polypropylene tubes and counted for 1 min with a Packard gamma counter. Specific binding was determined as the mean of quadruplicate samples of total binding minus the mean of quadruplicate nonspecific binding. The associated errors represent the square root of the sum of the standard deviations for total and nonspecific binding squared.

### Secreted alkaline phosphatase (SEAP) assay

GH4C1 cells transfected with α7 and NFκB SEAP were plated at 100, 000 cells/well in a 24-well plate in 0.5 ml complete medium (without antibiotics). On day 2, a 30-min pre-treatment was performed with either 100 μM PDTC or varying concentrations of GTS-21, followed by treatment with 20 ng/ml rat TNF. Cells without any treatment were used as negative controls. Twenty-four h later, 20 μl supernatant was collected from each well and mixed with 200 μl Invivogen SEAP substrate per manufacturer’s instructions to quantify relative SEAP secretion. Absorbance readings were taken at 630 nm.

### Statistical analysis

Quantitative data were expressed as mean ± standard error of the mean. Statistical analysis on ELISA data was conducted by one-way ANOVA followed by post-hoc Tukey HSD test to compare differences between multiple treatment groups. Student t-test was performed to analyze ^125^I-αBGT binding data.

## Results

### GTS-21 does not block NFκB-driven SEAP secretion in GH4C1 cells expressing α7 nAChR

**Fig 1:**
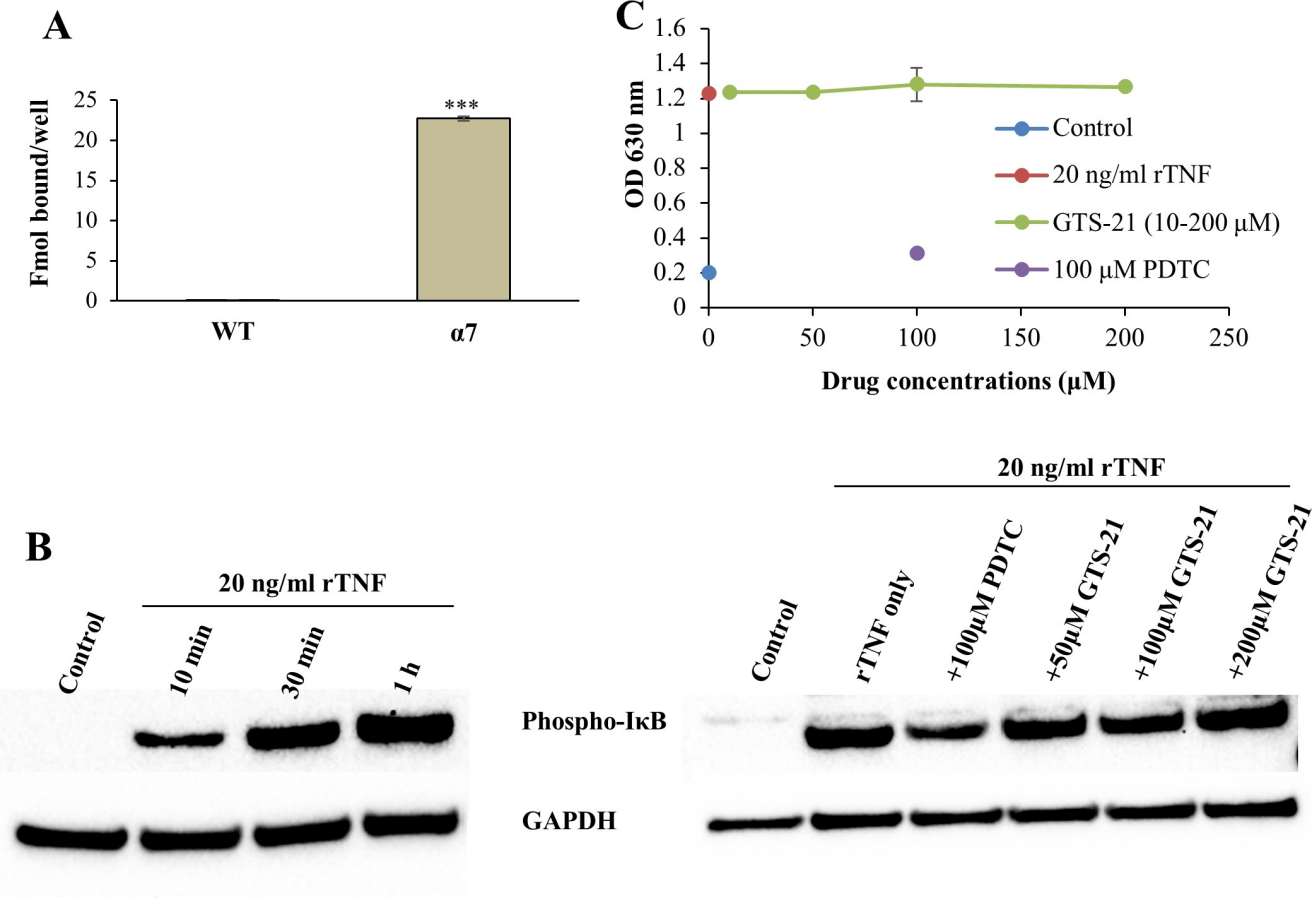
α7 nAChR expression and GTS-21 stimulation do not block TNF-induced NFκB-mediated signaling in GH4C1 cells. (A) ^125^I-αBGT binding to WT GH4C1 cells compared to α7 nAChR transfected cells (α7). Concentrations and error bars are as described in methods. *** P< 0.0001 compared to WT. (B) Left: Western blot data showing a time-dependent TNF-induced IκB phosphorylation levels. Right: Different concentrations of GTS-21 added 30 min prior to a 1 h TNF exposure (20 ng/ml), had no effect on pIκB levels in α7 nAChR transfected GH4C1 cells. In contrast, the NFκB blocker PDTC decreased the levels of pIκB. GAPDH is the loading control. (C) Effects of different concentrations of GTS-21 on TNF-induced SEAP. PDTC, an NFκB inhibitor, blocked TNF-induced SEAP, confirming that inflammatory signaling is intact in GH4C1 cells and that these cells can be used to study NFκB-mediated signaling. GTS-21 was unable to block TNF-induced SEAP in GH4C1 cells transfected with α7 nAChR and NFκB-SEAP plasmids.

GH4C1 cells transfected with α7 nAChR, but not WT cells, exhibit surface ^125^I-αBGT binding (Fig 1A). A 1 h treatment of cells transfected with both α7 nAChR and an NFκB-driven SEAP reporter gene with 20 ng/ml rat TNF (Fig 1B, left) causes robust phosphorylation of inhibitor of NFκB (IκB), an important inflammatory marker [6]. Briefly, TNF acting through its receptor activates Iκκ to eventually phosphorylate IκB, releasing NFκB. However, 30 min pre-treatment with various concentrations of GTS-21 did not change TNF-activated phospho-IκB levels, whereas 100 μM PDTC, an NFκB inhibitor that can block IkB degradation and phosphorylation [35, 36], partially blocked IκB phosphorylation (Fig 1B, right). TNF-mediated SEAP secretion instigates when TNF binds to TNF receptors to activate IκB through a cascade of downstream pathways. Phosphorylated IκB degrades, releasing NFκB which, when phosphorylated, dimerizes and translocates to the nucleus, where it acts as a transcription factor to bind a NFκB promoter sequence. Twenty ng/ml rat TNF at 24 h gave the optimal response for NFκB-driven SEAP secretion in GH4C1 cells transfected with the reporter gene (not shown). TNF-treated GH4C1 cells transfected with α7 nAChR and NFκB-SEAP showed a robust SEAP secretion into the cell medium, that acts as an indicator of NFκB activation, which was substantially inhibited by PDTC (Fig 1C). However, GTS-21 (10–200 μM) had no effect on TNF-driven SEAP secretion. Hence, GTS-21 inhibited neither TNF-dependent IκB phosphorylation nor TNF-dependent NFκB-driven SEAP secretion in GH4C1 cells expressing α7 nAChRs.

### Mouse macrophages express α7 nAChRs and GTS-21 blocks LPS-induced IκB phosphorylation

**Fig 2:**
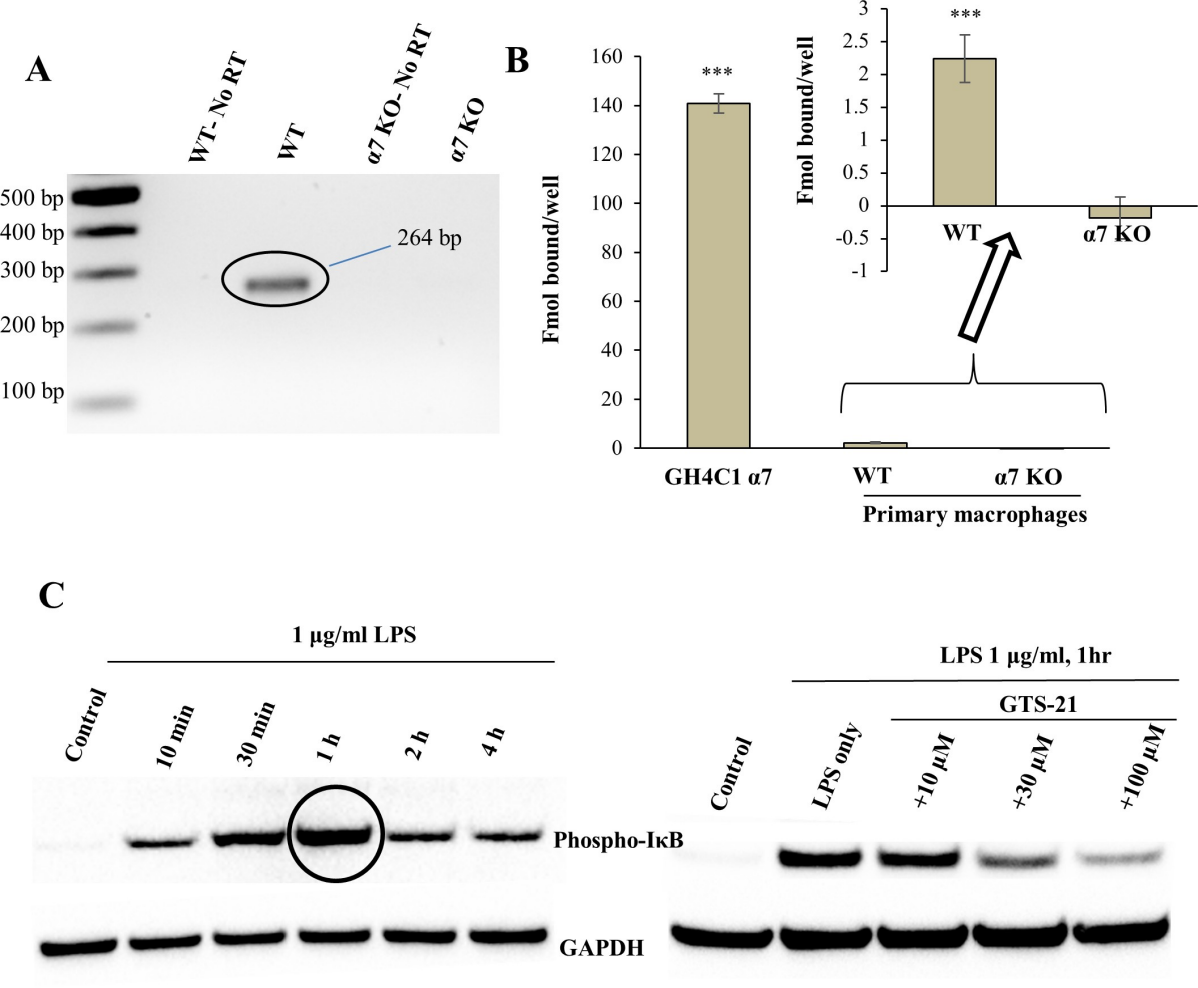
Confirmation of α7 nAChR surface expression on mouse macrophages. (A) PCR analysis showing a band of 264 bp size confirming the presence of α7 nAChR mRNA in macrophages isolated from WT but not α7 nAChR knockout mice. The 100 base pair ladder was from New England Biolabs. No RT: No reverse transcriptase control. (B) ^125^I-αBGT binding data comparing α7 nAChR expression on macrophages isolated from WT and α7 nAChR knockout murine model. α7 nAChR transfected GH4C1 cells were used as a positive control. Macrophages from WT mouse showed a small but significant αBGT specific binding site (detected with 100 nM ^125^I-αBGT). Bars in the figure represent specific binding. *** P< 0.0001 compared to α7 nAChR knockout mouse macrophages. (C) Left: Western blot data suggested LPS induced maximal pIκB levels in WT mouse macrophages at 1 h (circled). Right: GTS-21 attenuated LPS-stimulated pIκB levels in mouse macrophages in a dose-dependent manner. GAPDH was the loading control.

Primary macrophage cultures were stained with F4/80 and CD11b (Figure A in S1 File), two antibodies commonly used as macrophage markers, and over 90% of the cells stained with both markers. PCR analysis confirmed that α7 nAChR mRNA is expressed in macrophages from WT, but not *Chrna7*^-/-^ mice (Fig 2A). Sequencing confirmed the 264 bp PCR product identity. Binding studies corroborated these results, since WT macrophages showed specific αBGT binding, whereas no specific αBGT binding was observed in α7 nAChR knockout murine macrophages (Fig 2B). LPS induced significant levels of pIκB after 1 h treatment, and GTS-21 pre-treatment reduced LPS-induced pIκB in macrophages isolated from WT mice (Fig 2C).

### GTS-21 exhibits both α7 nAChR-mediated and α7 nAChR-independent anti-inflammatory effects in macrophages

**Fig 3:**
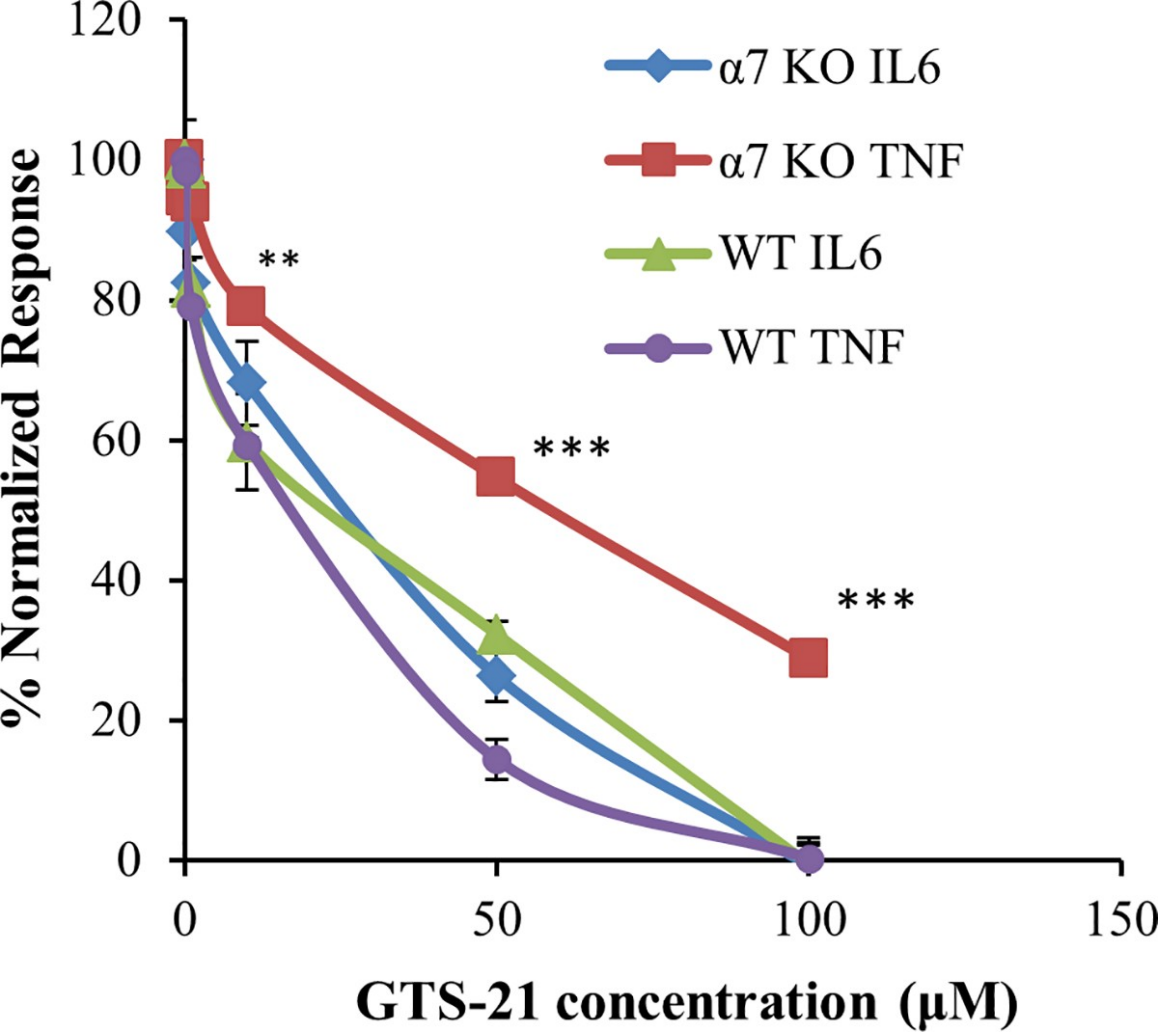
GTS-21 attenuates IL6 and TNF secretion in macrophages from WT and α7 nAChR knockout mice. Macrophages from WT and α7 nAChR knockout mice were isolated and activated by LPS before assaying the amount of IL6 and TNF release measured by ELISA as indicated in the methods section. Quadruplicate responses are normalized to cytokine release by LPS in the absence of GTS-21. A repetition of this experiment yielded similar results. One-way ANOVA (analysis of variance) with post-hoc Tukey HSD (honestly significant difference) test used to compare differences between multiple treatment groups. ** P< 0.01, *** P< 0.001 for the a7 KO TNF curve, other curves had similar P values (not shown).

**Fig 4:**
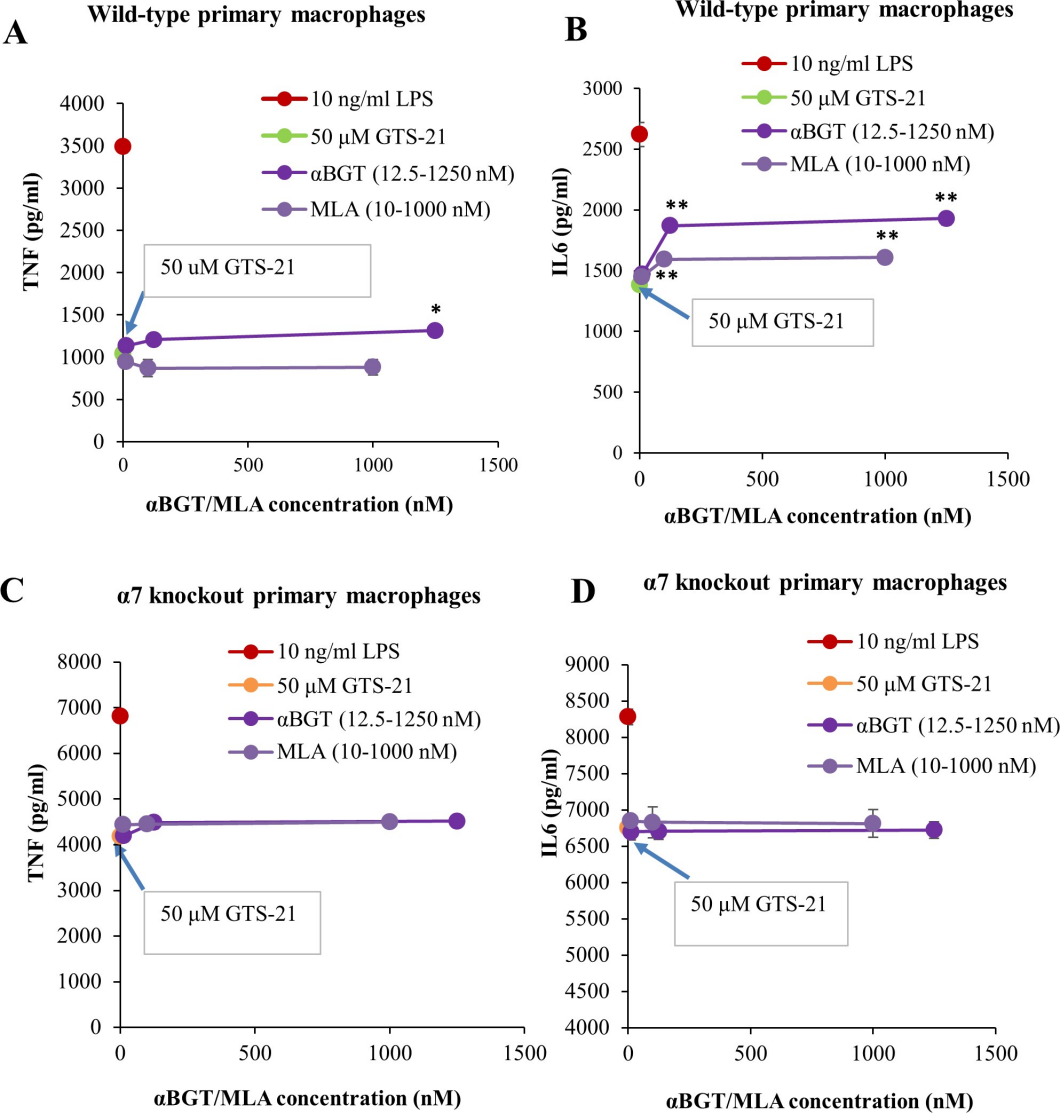
α7 nAChR-independent anti-inflammatory effects of GTS-21 in WT and *Chrna7*^*-/-*^ mouse macrophages. (A) 50 μM GTS-21 pre-treatment significantly blocked endotoxin-stimulated TNF and IL6 secretions and this concentration was selected to further study the effects of α7 nAChR antagonists, αBGT and MLA. ELISA data showing approx. 10% reversal of GTS-21 block on TNF secretion by 1.25 μM αBGT. No reversal was observed with MLA. ELISA data in panel (B) demonstrates 40% reversal of GTS-21 block on IL6 secretion by 1.25 μM αBGT and 30% reversal by 1 μM MLA. Neither αBGT nor MLA produced significant changes in the GTS-21 block of LPS-induced (C) TNF or (D) IL6 secretion in α7 nAChR absence. One-way ANOVA (analysis of variance) with post-hoc Tukey HSD (honestly significant difference) test used to compare differences between multiple treatment groups. * P< 0.01, ** P< 0.001, *** P< 0.0001. A duplicate of this experiment showed similar effects.

Primary mouse macrophages from both WT and α7 nAChR knockout mice showed robust TNF and IL6 secretion in response to LPS treatment. Ten ng/ml LPS applied to WT mouse macrophages gave submaximal but significant IL6 and TNF secretion, and this LPS concentration was used in subsequent experiments. GTS-21 dose-dependently attenuated TNF and IL6 secretion in macrophages isolated from both WT and α7 nAChR knockout mice, confirming that GTS-21 possess α7 nAChR-independent anti-inflammatory effects (Fig 3). This effect was not due to gross changes in cell viability (Figure B in S1 File). Fifty μM GTS-21 pre-treatment reduced >50% cytokine response in mouse macrophages and was used to perform further studies using α7 nAChR antagonists (αBGT and MLA) [37]. The α7 nAChR antagonist αBGT showed about 10% reversal of GTS-21 induced block of TNF release by LPS in macrophages isolated from WT mice, but MLA elicited no such reversal (Fig 4A). Additionally, αBGT reversed about 40%, and MLA about 30% of the GTS-21 block of IL6 release by LPS in WT macrophages (Fig 4B). The partial αBGT and MLA reversals of GTS-21 blockade suggest some α7 nAChR-dependent anti-inflammatory effects of GTS-21. However, GTS-21 blocked TNF and IL6 secretion in α7 nAChR knockout macrophages, and neither αBGT nor MLA has any effect on either TNF or IL6 secretion (Fig 4C & 4D). These data strongly suggest that GTS-21 mediates anti-inflammatory effects in mouse macrophages at least in part via an α7 nAChR-independent pathway.

## Discussion

Chronic inflammation often occurs due to either unresolved injuries or pathogenic infections that cause over-production of multiple pro-inflammatory cytokines such as TNF, IL1, IL6, IL17, HMGB1 and other mediators such as nitric oxide. Monoclonal antibodies are used clinically to prevent inflammation associated with various disease states by binding to specific pro-inflammatory cytokines. An example is etanercept, a TNF antibody used to treat rheumatoid arthritis [38]. However, a major challenge has been to find orally bioavailable small molecules that simultaneously suppress multiple pro-inflammatory cytokines. Wang et al. [7] reported that vagus nerve stimulation activates cholinergic signaling via α7 nAChRs to inhibit chronic inflammation. Since then, many reports (based on *in vivo* and *in vitro* experiments) have shown that like vagus nerve stimulation, α7 nAChR agonists inhibit multiple pro-inflammatory cytokines [10, 17]. GTS-21 is an α7 nAChR partial agonist commonly used as an anti-inflammatory agent [24–27]. In this report, we investigated whether α7 nAChRs are required for GTS-21’s proposed anti-inflammatory effects, both in immune-derived cells and in a heterologous expression model in a non-immune cell system.

GH4C1 cells, derived from rat pituitary do not express any known nicotinic receptor subunits, while α7 nAChRs heterologously expressed in GH4C1 cells [29] show classic properties of α7 ligand-gated ion channels. Activation with agonist causes cell depolarizations that desensitize rapidly [30] show high calcium permeability [39, 40] and even mutant α7 receptors can show single channel currents as well as inward rectification [41]. Receptor activation in GH4C1 cells is blocked by α7 antagonists such as MLA [30], and agonist-induced ion channel activity can be dramatically increased by α7 positive allosteric modulators such as PNU-120596 [40]. The concentration of MLA necessary to block α7 nAChRs is 1 nM in GH4C1 cells [30], while that of αBGT in other heterologous expression systems is around 10 nM [42, 43]. Therefore, it is a reasonable assumption that appearance of αBGT binding sites after transfection of α7 nAChR DNA in GH4C1 cells correlates with expression of electrophysiologically-functional α7 nAChRs ion channels. In contrast, nAChRs expressed on myeloid immune-derived cell systems have to date shown no evidence of electrophysiological function. Nicotine blocked TNF release in rat microglia via intracellular calcium release in the absence of cell surface ion channel function, but the effect was blocked by 10 nM αBGT or MLA, suggesting involvement of α7 nAChRs [44]. Similarly, nicotine, acetylcholine, choline, and several choline derivatives effectively blocked ATP-induced release of the pro-inflammatory cytokine IL-1β from rat and human monocytes expressing α7, α9 and/or α10 subunits, but nicotine induced no current in these cells, unlike ATP [45]. In those monocyte cultures, 1 μM αBGT blocked the effects. Also, nicotine blocked the ability of ATP to increase intracellular calcium in rat alveolar macrophages expressing α9 and α10 subunits, and 100 nM αBGT blocked the effects, but no nicotinic channel function was observed, and extracellular calcium was not required for these effects [46]. Thomsen and Mikkelsen [47] demonstrated that the weak partial α7 agonists GTS-21 (50–100 μM) and NS6740 (50 μM) and the α7 antagonist MLA (10 nM) significantly attenuated LPS-induced TNF release from cultured rat microglia, while adding positive allosteric modulators such as PNU120596 had no further effect. Together, these results have led to the hypothesis that ionotropic channel action is not required for nicotinic receptor anti-inflammatory actions in myeloid tissues, and that “silent agonists” can bind to α7 nAChRs to produce metabotropic-like activity that is responsible for anti-inflammatory actions [48]. The basis for functional differences between nicotinic receptors expressed in myeloid tissues and neuronal tissues is not known.

**Fig 5:**
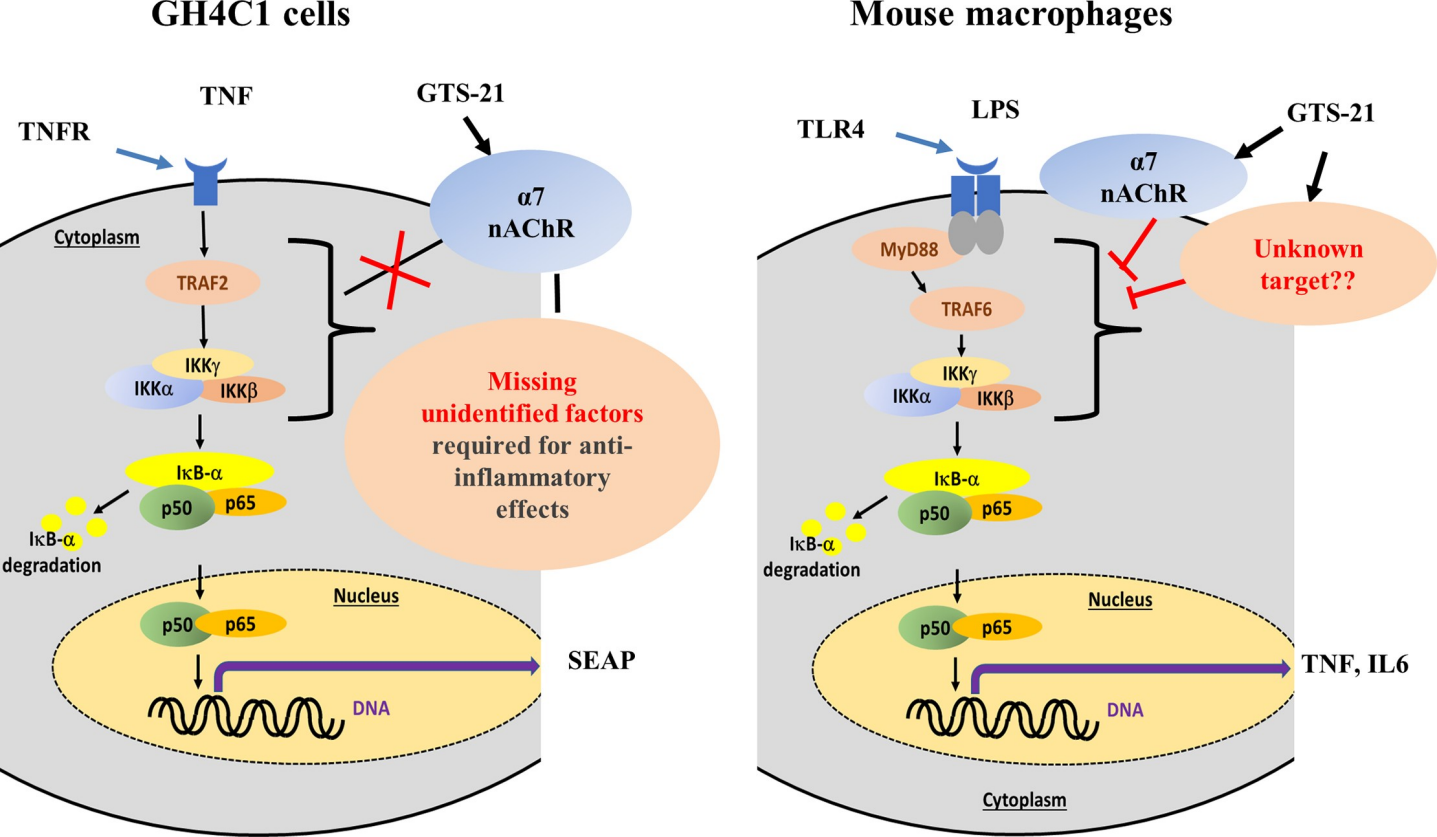
Schematic comparing cell-dependent effects of GTS-21 in GH4C1 cells and primary mouse macrophages. Top panel: GTS-21 was unable to suppress TNF-induced secreted alkaline phosphatase in a α7 nAChR and NFκB-SEAP expressing GH4C1 cell system. Bottom panel: GTS-21 reduced LPS-induced TNF and IL6 levels through both an α7 nAChR-dependent and an α7 nAChR-independent mechanism in primary mouse macrophages. In both cases, activation of the NFkB pathway leads to phosphorylation of IkB, and dimerization and translocation of two subunits of cytoplasmic NFκB (usually p50 and p65) to the nucleus to act as a transcription factor (Lawrence et al., 2009). In spite of the common NFkB pathway, the immediate effects of TNF and LPS differ in terms of the receptors and linkage to NFkB signaling, where GTS-21 non-nicotinic receptor actions may be taking place.

We used GH4C1 cells expressing both α7 nAChR and the gene reporter NFκB-SEAP (Fig 1) to check whether α7 nAChR expression alters GTS-21 effects on NFκB signaling. ^125^I-αBGT binding studies validated that α7 nAChR are being expressed, while SEAP secretion assays and western blots of IκBα phosphorylation measured rat TNF-activated NFκB signaling. However, GTS-21 (100 nM—200 μM) had no blocking effect on TNF effects. These data suggest that GTS-21 is unable to alter TNF signaling by activating α7 nAChR ion channels in GH4C1 cells, unlike the GTS-21 blockade we observed for LPS-induced signaling in macrophages. This could be due to differences in downstream inflammatory signaling between TNF receptors and LPS-activated TLR4 receptors. TNF receptors signal through TNF receptor associated factor 2 (TRAF2) and other components upstream of NFκB, while LPS signals through myeloid differentiation primary response gene 88 (MyD88) and TRAF6, among others (Fig 5, [6]). GTS-21 may not affect TNF-induced inflammatory signaling in GH4C1 cells via α7 nAChR, as it may need adaptor molecules like MyD88 and TRAF6. We hypothesize that GTS-21 effects on NFκB signaling may require such adaptor molecules and additional unidentified factors which are not present in non-immune GH4C1 cells. It is possible that the same unidentified factors required to link α7 receptors to dampen inflammatory signaling also silence the ion channel properties of the receptors in myeloid tissue.

Wang et al.[7]showed that human macrophages possess α7 nAChR RNA signal by PCR analysis and FITC-labeled αBGT staining. Similarly, we found subtle but significant α7 nAChR RNA expression and ^125^I-αBGT specific binding in macrophages isolated from WT mice, but both were absent in α7 nAChR knockout mouse macrophages. Wang et al. [7] used the α7 nAChR agonist nicotine, to demonstrate α7 nAChR-mediated anti-inflammatory effects on LPS-induced cytokines secretion by comparing the effects in WT and α7 nAChR knockout mouse macrophages. However, nicotine toxicity, i.e., nausea, headache, and addiction, has raised concerns over the specificity of anti-inflammatory effects of nicotine [22, 49], and has raised the question of whether more selective α7 nAChR agonists, such as GTS-21, should be substituted to achieve anti-inflammatory effects.

GTS-21 blocks LPS-induced cytokine RNA levels [27, 50, 51] and LPS-driven IL1, IL6, and TNF release [27] measured by ELISA in leukocyte or macrophage cultures or in whole animals. Likewise, in our experiments, 50 μM GTS-21 blocked >50% LPS-induced pIκB levels and TNF and IL6 secretion. Nullens et al. [28] studied a murine cecal ligation and puncture-induced sepsis model and found that GTS-21 reduced colon and serum IL6 levels in both WT and α7 nAChR knockout animals. In contrast, Rosas-Ballina et al. [50] reported that GTS-21 alleviates LPS-induced inflammatory signaling in macrophages via α7 nAChRs. Our results establish that GTS-21 retains its anti-inflammatory effects at the cellular level, inhibiting TNF and IL6 secretion in macrophages from the *Chrna7*^-/-^ mouse. Moreover, α7 nAChR antagonists, MLA and αBGT, partially reversed GTS-21 blockade of LPS-induced TNF and IL6 secretion, but only in macrophages isolated from WT mice and had no effect on α7 nAChR knockout macrophages. Further, the concentrations of these antagonists we used were well in excess of concentrations necessary to block function at ionotropic α7 nAChRs, and the maximum effects were very modest. These *ex vivo* data confirm that GTS-21 has both α7 nAChR-mediated and α7 nAChR-independent anti-inflammatory effects. GTS-21 might produce α7 nAChR-independent anti-inflammatory effects via other nicotinic subtypes like α4β2 [52], as some reports claim that α4β2 nAChRs also play a crucial role in suppressing inflammation [53, 54]. Another report indicates that the nAChR agonist anatabine exhibits α7 nAChR-independent anti-inflammatory effects in HEK-293 cells that lack nAChRs [55].

In summary, GTS-21 effects are highly cell-type dependent, and the presence of α7 nAChR is insufficient to allow GTS-21 to block NFκB signaling in all cell types. Further, GTS-21 can block NFκB signaling in cells lacking α7 nAChRs. Thus, anti-inflammatory effects of GTS-21 should not necessarily be ascribed to its effects as a partial α7 nAChR agonist.

## Supporting information

S1 File Supplementary methods and figures. (PDF)

## References

[1] Heap GA, van Heel DA. The genetics of chronic inflammatory diseases. Hum Mol Genet. 2009;18: R101–106. 10.1093/hmg/ddp001 19297396

[2] Rimmer E, Houston BL, Kumar A, Abou-Setta AM, Friesen C, Marshall JC, et al The efficacy and safety of plasma exchange in patients with sepsis and septic shock: a systematic review and meta-analysis. Crit Care. 2014;18: 699 10.1186/s13054-014-0699-2 25527094 PMC4318234

[3] de Jong PR, Gonzalez-Navajas JM, Jansen NJ. The digestive tract as the origin of systemic inflammation. Crit Care. 2016;20: 279 10.1186/s13054-016-1458-3 27751165 PMC5067918

[4] Koopman FA, Chavan SS, Miljko S, Grazio S, Sokolovic S, Schuurman PR, et al Vagus nerve stimulation inhibits cytokine production and attenuates disease severity in rheumatoid arthritis. Proc Natl Acad Sci U S A. 2016;113: 8284–8289. 10.1073/pnas.1605635113 27382171 PMC4961187

[5] Borovikova LV, Ivanova S, Zhang M, Yang H, Botchkina GI, Watkins LR, et al Vagus nerve stimulation attenuates the systemic inflammatory response to endotoxin. Nature. 2000;405: 458–462. 10.1038/35013070 10839541

[6] Viatour P, Merville MP, Bours V, Chariot A. Phosphorylation of NF-kappaB and IkappaB proteins: implications in cancer and inflammation. Trends Biochem Sci. 2005;30: 43–52. 10.1016/j.tibs.2004.11.009 15653325

[7] Wang H, Yu M, Ochani M, Amella CA, Tanovic M, Susarla S, et al Nicotinic acetylcholine receptor alpha7 subunit is an essential regulator of inflammation. Nature. 2003;421: 384–388. 10.1038/nature01339 12508119

[8] Wang H, Liao H, Ochani M, Justiniani M, Lin X, Yang L, et al Cholinergic agonists inhibit HMGB1 release and improve survival in experimental sepsis. Nat Med. 2004;10: 1216–1221. 10.1038/nm1124 15502843

[9] Andersson U, Tracey KJ. Reflex principles of immunological homeostasis. Annu Rev Immunol. 2012;30: 313–335. 10.1146/annurev-immunol-020711-075015 22224768 PMC4533843

[10] de Jonge WJ, Ulloa L. The alpha7 nicotinic acetylcholine receptor as a pharmacological target for inflammation. Br J Pharmacol. 2007;151: 915–929. 10.1038/sj.bjp.0707264 17502850 PMC2042938

[11] Banerjee C, Nyengaard JR, Wevers A, de Vos RA, Jansen Steur EN, Lindstrom J, et al Cellular expression of alpha7 nicotinic acetylcholine receptor protein in the temporal cortex in Alzheimer's and Parkinson's disease—a stereological approach. Neurobiol Dis. 2000;7: 666–672. 10.1006/nbdi.2000.0317 11114264

[12] Leonard S, Adams C, Breese CR, Adler LE, Bickford P, Byerley W, et al Nicotinic receptor function in schizophrenia. Schizophr Bull. 1996;22: 431–445. 8873294 10.1093/schbul/22.3.431

[13] Levin ED. alpha7-Nicotinic receptors and cognition. Curr Drug Targets. 2012;13(5): 602–606. 22300026 10.2174/138945012800398937

[14] Maouche K, Medjber K, Zahm JM, Delavoie F, Terryn C, Coraux C, et al Contribution of alpha7 nicotinic receptor to airway epithelium dysfunction under nicotine exposure. Proc Natl Acad Sci U S A. 2013;110: 4099–4104. 10.1073/pnas.1216939110 23431157 PMC3593882

[15] Quik M, Zhang D, McGregor M, Bordia T. Alpha7 nicotinic receptors as therapeutic targets for Parkinson's disease. Biochem Pharmacol. 2015;97: 399–407. 10.1016/j.bcp.2015.06.014 26093062 PMC4600450

[16] Nizri E, Brenner T. Modulation of inflammatory pathways by the immune cholinergic system. Amino acids. 2013;45: 73–85. 10.1007/s00726-011-1192-8 22194043

[17] van Maanen MA, Lebre MC, van der Poll T, LaRosa GJ, Elbaum D, Vervoordeldonk MJ, et al Stimulation of nicotinic acetylcholine receptors attenuates collagen-induced arthritis in mice. Arthritis Rheum. 2009;60: 114–122. 10.1002/art.24177 19116908

[18] Matsunaga K, Klein TW, Friedman H, Yamamoto Y. Involvement of nicotinic acetylcholine receptors in suppression of antimicrobial activity and cytokine responses of alveolar macrophages to Legionella pneumophila infection by nicotine. J Immunol. 2001;167: 6518–6524. 11714820 10.4049/jimmunol.167.11.6518

[19] Pullan RD, Rhodes J, Ganesh S, Mani V, Morris JS, Williams GT, et al Transdermal nicotine for active ulcerative colitis. N Engl J Med. 1994;330: 811–815. 10.1056/NEJM199403243301202 8114833

[20] Sykes AP, Brampton C, Klee S, Chander CL, Whelan C, Parsons ME. An investigation into the effect and mechanisms of action of nicotine in inflammatory bowel disease. Inflamm Res. 2000;49: 311–319. 10.1007/s000110050597 10959551

[21] Wittebole X, Hahm S, Coyle SM, Kumar A, Calvano SE, Lowry SF. Nicotine exposure alters in vivo human responses to endotoxin. Clin Exp Immunol. 2007;147: 28–34. 10.1111/j.1365-2249.2006.03248.x 17177960 PMC1810444

[22] Ingram JR, Routledge P, Rhodes J, Marshall RW, Buss DC, Evans BK, et al Nicotine enemas for treatment of ulcerative colitis: a study of the pharmacokinetics and adverse events associated with three doses of nicotine. Aliment Pharmacol Ther. 2004;20: 859–865. 10.1111/j.1365-2036.2004.02199.x 15479357

[23] Mishra A, Chaturvedi P, Datta S, Sinukumar S, Joshi P, Garg A. Harmful effects of nicotine. Indian J Med Paediatr Oncol. 2015;36: 24–31. 10.4103/0971-5851.151771 25810571 PMC4363846

[24] Pavlov VA, Ochani M, Yang LH, Gallowitsch-Puerta M, Ochani K, Lin X, et al Selective alpha7-nicotinic acetylcholine receptor agonist GTS-21 improves survival in murine endotoxemia and severe sepsis. Crit Care. 2007;35: 1139–1144. 10.1097/01.Ccm.0000259381.56526.96 17334244

[25] Chatterjee PK, Yeboah MM, Dowling O, Xue X, Powell SR, Al-Abed Y, et al Nicotinic acetylcholine receptor agonists attenuate septic acute kidney injury in mice by suppressing inflammation and proteasome activity. PLoS One. 2012;7: e35361 10.1371/journal.pone.0035361 22586448 PMC3346807

[26] Khan MA, Farkhondeh M, Crombie J, Jacobson L, Kaneki M, Martyn JA. Lipopolysaccharide upregulates alpha7 acetylcholine receptors: stimulation with GTS-21 mitigates growth arrest of macrophages and improves survival in burned mice. Shock. 2012;38: 213–219. 10.1097/SHK.0b013e31825d628c 22683726 PMC3399057

[27] Kox M, van Velzen JF, Pompe JC, Hoedemaekers CW, van der Hoeven JG, Pickkers P. GTS-21 inhibits pro-inflammatory cytokine release independent of the Toll-like receptor stimulated via a transcriptional mechanism involving JAK2 activation. Biochem Pharmacol. 2009;78: 863–872. 10.1016/j.bcp.2009.06.096 19576181

[28] Nullens S, Staessens M, Peleman C, Schrijvers DM, Malhotra-Kumar S, Francque S, et al Effect of GTS-21, an alpha7 nicotinic acetylcholine receptor agonist, on CLP-induced inflammatory, gastrointestinal motility, and colonic permeability changes in mice. Shock. 2016;45: 450–459. 10.1097/SHK.0000000000000519 26618987

[29] Quik M, Choremis J, Komourian J, Lukas RJ, Puchacz E. Similarity between rat brain nicotinic alpha-bungarotoxin receptors and stably expressed alpha-bungarotoxin binding sites. J Neurochem. 1996;67: 145–154. 8666985 10.1046/j.1471-4159.1996.67010145.x

[30] Virginio C, Giacometti A, Aldegheri L, Rimland JM, Terstappen GC. Pharmacological properties of rat alpha 7 nicotinic receptors expressed in native and recombinant cell systems. Eur J Pharmacol. 2002;445: 153–161. 12079679 10.1016/s0014-2999(02)01750-8

[31] Zhang X, Goncalves R, Mosser DM. The isolation and characterization of murine macrophages. Curr Protoc Immunol. 2008;Chapter 14:Unit 14.1. 10.1002/0471142735.im1401s83 19016445 PMC2834554

[32] Schulz DW, Loring RH, Aizenman E, Zigmond RE. Autoradiographic localization of putative nicotinic receptors in the rat brain using 125I-neuronal bungarotoxin. J Neurosci. 1991;11: 287–297. 1986068 10.1523/JNEUROSCI.11-01-00287.1991 PMC6575193

[33] Garg BK, Loring RH. Evaluating Commercially Available Antibodies for Rat alpha7 Nicotinic Acetylcholine Receptors. J Histochem Cytochem. 2017;65: 499–512. 10.1369/0022155417725304 28763248 PMC5582671

[34] Koperniak TM, Garg BK, Boltax J, Loring RH. Cell-specific effects on surface alpha7 nicotinic receptor expression revealed by over-expression and knockdown of rat RIC3 protein. J Neurochem. 2013;124: 300–309. 10.1111/jnc.12095 23157401

[35] Cuzzocrea S, Chatterjee PK, Mazzon E, Dugo L, Serraino I, Britti D, et al Pyrrolidine dithiocarbamate attenuates the development of acute and chronic inflammation. Br J Pharmacol. 2002;135: 496–510. 10.1038/sj.bjp.0704463 11815386 PMC1573136

[36] Zhang JJ, Xu ZM, Zhang CM, Dai HY, Ji XQ, Wang XF, et al Pyrrolidine dithiocarbamate inhibits nuclear factor-kappaB pathway activation, and regulates adhesion, migration, invasion and apoptosis of endometriotic stromal cells. Mol Hum Reprod. 2011;17: 175–181. 10.1093/molehr/gaq090 21030494

[37] Zhao L, Kuo YP, George AA, Peng JH, Purandare MS, Schroeder KM, et al Functional properties of homomeric, human alpha 7-nicotinic acetylcholine receptors heterologously expressed in the SH-EP1 human epithelial cell line. J Pharmacol Exp Ther. 2003;305: 1132–1141. 10.1124/jpet.103.048777 12626641

[38] Weinblatt ME, Bathon JM, Kremer JM, Fleischmann RM, Schiff MH, Martin RW, et al Safety and efficacy of etanercept beyond 10 years of therapy in North American patients with early and longstanding rheumatoid arthritis. Arthritis Care Res. 2011;63: 373–382. 10.1002/acr.20372 20957659

[39] Quik M, Philie J, Choremis J. Modulation of alpha7 nicotinic receptor-mediated calcium influx by nicotinic agonists. Mol Pharm. 1997;51: 499–506. 9058606

[40] Dunlop J, Lock T, Jow B, Sitzia F, Grauer S, Jow F, et al Old and new pharmacology: positive allosteric modulation of the alpha7 nicotinic acetylcholine receptor by the 5-hydroxytryptamine(2B/C) receptor antagonist SB-206553 (3,5-dihydro-5-methyl-N-3-pyridinylbenzo[1,2-b:4,5-b']di pyrrole-1(2H)-carboxamide). J Pharmacol Exp Ther. 2009;328: 766–776. 10.1124/jpet.108.146514 19050173

[41] Placzek AN, Grassi F, Meyer EM, Papke RL. An alpha7 nicotinic acetylcholine receptor gain-of-function mutant that retains pharmacological fidelity. Mol Pharm. 2005;68: 1863–1876. 10.1124/mol.105.016402 16186249

[42] Seguela P, Wadiche J, Dineley-Miller K, Dani JA, Patrick JW. Molecular cloning, functional properties, and distribution of rat brain alpha 7: a nicotinic cation channel highly permeable to calcium. J Neurosci. 1993;13: 596–604. 7678857 10.1523/JNEUROSCI.13-02-00596.1993 PMC6576637

[43] Peng X, Katz M, Gerzanich V, Anand R, Lindstrom J. Human alpha 7 acetylcholine receptor: cloning of the alpha 7 subunit from the SH-SY5Y cell line and determination of pharmacological properties of native receptors and functional alpha 7 homomers expressed in Xenopus oocytes. Mol Pharm. 1994;45: 546–554. 8145738

[44] Suzuki T, Hide I, Matsubara A, Hama C, Harada K, Miyano K, et al Microglial alpha7 nicotinic acetylcholine receptors drive a phospholipase C/IP3 pathway and modulate the cell activation toward a neuroprotective role. J Neurosci Res. 2006;83: 1461–1470. 10.1002/jnr.20850 16652343

[45] Hecker A, Kullmar M, Wilker S, Richter K, Zakrzewicz A, Atanasova S, et al Phosphocholine-Modified Macromolecules and Canonical Nicotinic Agonists Inhibit ATP-Induced IL-1beta Release. J Immunol. 2015;195: 2325–2334. 10.4049/jimmunol.1400974 26202987

[46] Mikulski Z, Hartmann P, Jositsch G, Zaslona Z, Lips KS, Pfeil U, et al Nicotinic receptors on rat alveolar macrophages dampen ATP-induced increase in cytosolic calcium concentration. Respir Res. 2010;11: 133 10.1186/1465-9921-11-133 20920278 PMC2955664

[47] Thomsen MS, Mikkelsen JD. The alpha7 nicotinic acetylcholine receptor ligands methyllycaconitine, NS6740 and GTS-21 reduce lipopolysaccharide-induced TNF-alpha release from microglia. J Neuroimmunol. 2012;251: 65–72. 10.1016/j.jneuroim.2012.07.006 22884467

[48] Horenstein NA, Papke RL. Anti-inflammatory Silent Agonists. ACS Med Chem Lett. 2017;8: 989–991. 10.1021/acsmedchemlett.7b00368 29057037 PMC5641964

[49] Ulloa L. The vagus nerve and the nicotinic anti-inflammatory pathway. Nat Rev Drug Discov. 2005;4: 673–684. 10.1038/nrd1797 16056392

[50] Rosas-Ballina M, Goldstein RS, Gallowitsch-Puerta M, Yang L, Valdes-Ferrer SI, Patel NB, et al The selective alpha7 agonist GTS-21 attenuates cytokine production in human whole blood and human monocytes activated by ligands for TLR2, TLR3, TLR4, TLR9, and RAGE. Molecular medicine (Cambridge, Mass). 2009;15: 195–202. 10.2119/molmed.2009.00039 19593403 PMC2707516

[51] Yang X, Zhao C, Chen X, Jiang L, Su X. Monocytes primed with GTS-21/alpha7 nAChR (nicotinic acetylcholine receptor) agonist develop anti-inflammatory memory. Qjm 2017;110: 437–445. E 10.1093/qjmed/hcx014 28082382

[52] Meyer EM, Kuryatov A, Gerzanich V, Lindstrom J, Papke RL. Analysis of 3-(4-hydroxy, 2-Methoxybenzylidene)anabaseine selectivity and activity at human and rat alpha-7 nicotinic receptors. J Pharmacol Exp Ther. 1998;287: 918–925. 9864273

[53] Hosur V, Loring RH. alpha4beta2 nicotinic receptors partially mediate anti-inflammatory effects through Janus kinase 2-signal transducer and activator of transcription 3 but not calcium or cAMP signaling. Mol Pharm. 2011;79: 167–174. 10.1124/mol.110.066381 20943775

[54] van der Zanden EP, Snoek SA, Heinsbroek SE, Stanisor OI, Verseijden C, Boeckxstaens GE, et al Vagus nerve activity augments intestinal macrophage phagocytosis via nicotinic acetylcholine receptor alpha4beta2. Gastroenterology. 2009;137(3): 1029–39, 39.e1-4. Epub 2009/05/12. 10.1053/j.gastro.2009.04.057 . 19427310

[55] Paris D, Beaulieu-Abdelahad D, Bachmeier C, Reed J, Ait-Ghezala G, Bishop A, et al Anatabine lowers Alzheimer's Abeta production in vitro and in vivo. Eur J Pharmacol. 2011;670: 384–391. 10.1016/j.ejphar.2011.09.019 21958873

