# Supplementary material for: GTS-21 has cell-specific anti-inflammatory effects independent of α7 nicotinic acetylcholine receptors
Source: PLoS One. 2019 Apr 4;14(4):e0214942. doi: 10.1371/journal.pone.0214942 (PMC6448884; doi:10.1371/journal.pone.0214942)
Supplement: S1 File — (PDF) [file pone.0214942.s001.pdf]

Supplementary file S1.

GTS-21 has cell-specific anti-inflammatory effects independent of α7 nicotinic acetylcholine receptors

Brijesh K. Garg and Ralph H. Loring

Supplementary methods:

Immunofluorescence

Cells were plated on 10 µg/cm^2 poly-L-lysine pre-coated sterile coverslips placed in 24-multiwell plates. Cells were washed the next day with 1 ml PBS/well, fixed for 15 min in 4% formaldehyde in PBS and permeabilized in 0.4% triton X-100 in PBS. Cells were then washed 3-5 times in ice-cold PBS before blocking with 2% BSA for 1 h at room temperature to minimize non-specific binding. F4/80 and CD11b antibodies bound overnight at dilutions of 1:500 in blocking buffer, and then were washed away three times with 1 ml ice-cold PBS. Coverslips were mounted on glass slides using mounting medium (Sigma-Aldrich Fluoroshield, catalog# F6057) and left overnight at 4°C in the dark before microscopic examination.

Supplementary figures:

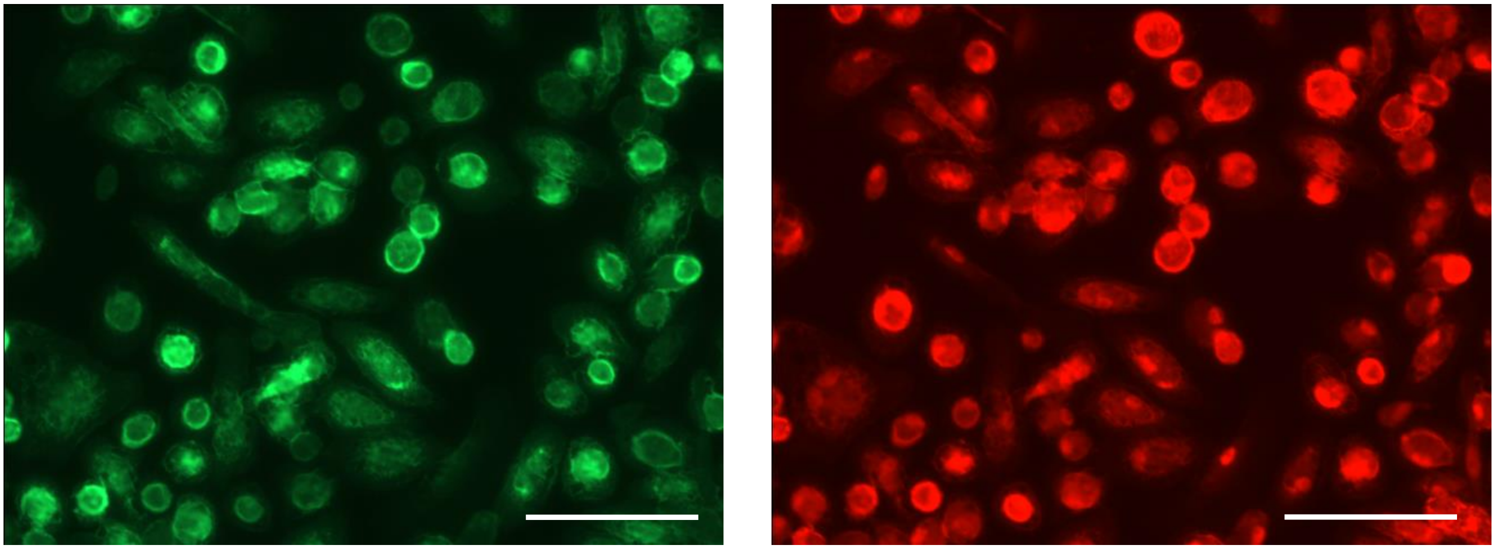

CD11b F4/80

S1 Figure A.

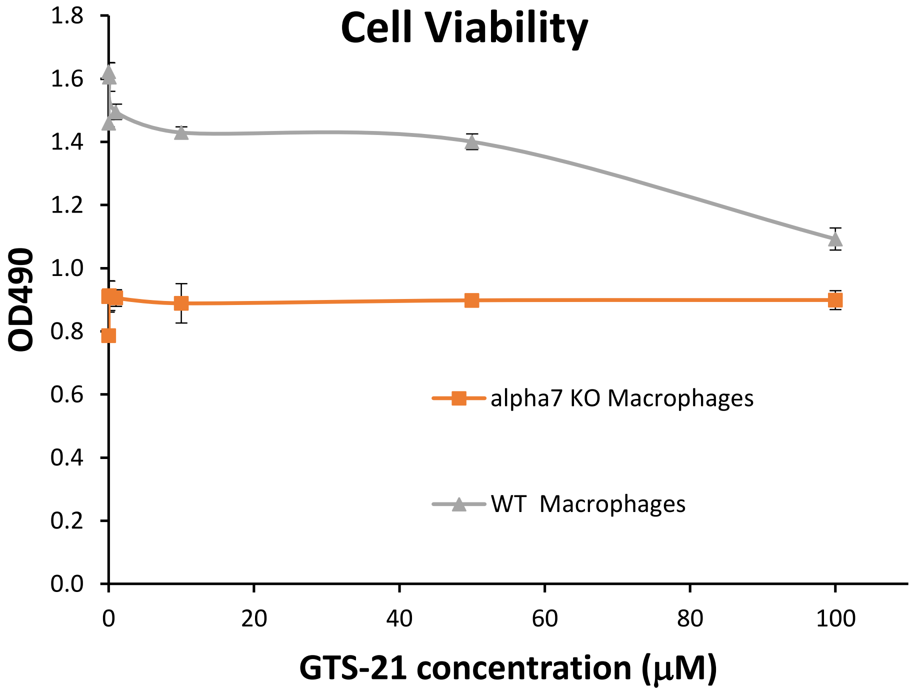
Cell Viability
OD490
1.8
1.6
1.4
1.2
1.0
0.8
0.6
0.4
0.2
0.0
0
20
40
60
80
100
GTS-21 concentration (μM)
alpha7 KO Macrophages
WT Macrophages

S1 Figure B.
